# Supplementary material for: Adaptive plasticity of aspartate metabolism in succinate dehydrogenase-deficient cancer cells
Source: bioRxiv. 2026 Jun 11:2026.05.18.726122. Originally published 2026 May 21. Preprint. [Version 2] doi: 10.64898/2026.05.18.726122 (PMC13228284; doi:10.64898/2026.05.18.726122)
Supplement: Supplement 1 [file media-1.pdf]

Supplemental information for: adaptive plasticity of aspartate metabolism in succinate dehydrogenase-deficient cancer cells

David Sokolov[1]**, Eric Zheng**[1]**, Serwah Danquah**[1]**, Madeleine L. Hart**[1]**, and Lucas B. Sullivan**[1,2]

[1] Human Biology Division, Fred Hutchinson Cancer Center, Seattle, WA, 98109, USA.

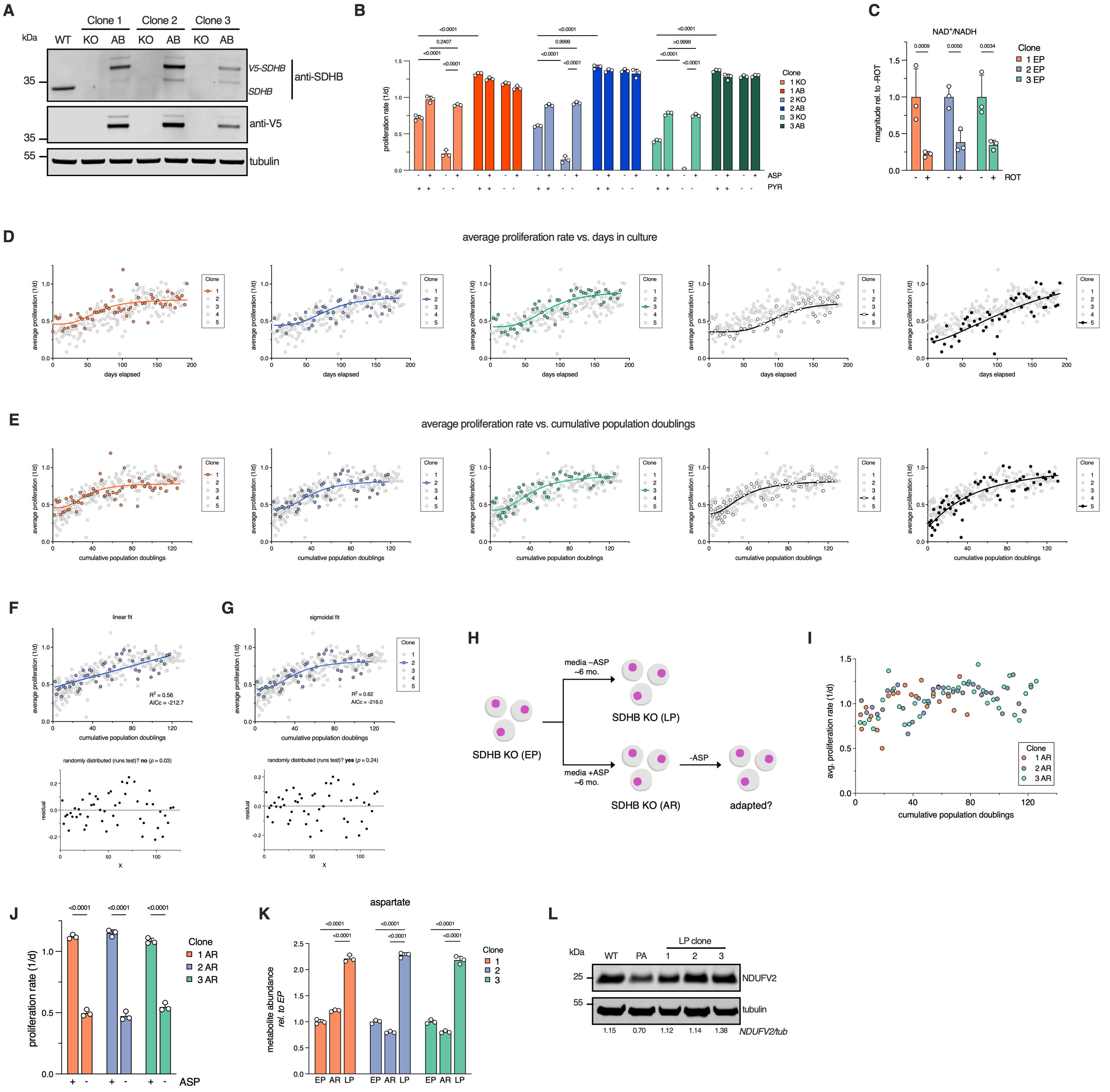

Fig. S1. Related to Figure 1. (**A**) Representative western blot showing levels of SDHB, V5-tagged SDHB, and tubulin loading control in wild-type parental cells (WT), early passage (EP), and addback (AB) SDHB knockout clones 1-3. (**B**) Absolute proliferation rates (mean +/- S.D.) of early passage (EP) and addback (AB) clones 1-3 in DMEM with all combinations of 1 mM pyruvate and 20 mM aspartate supplementation. Statistical significance determined using an ordinary two-way ANOVA and Tukey's multiple comparisons test with a single pooled variance. Data for KO clones in pyruvate-containing media are the same as plotted in Figure 1H. (n=3) (**C**) Relative whole-cell $NAD^+/NADH$ (mean +/- S.D.), measured using LCMS, in SDHB-KO clones 1-3 after 6 hours of treatment with vehicle control or 50 nM rotenone (ROT). Values are normalized to the vehicle-treated condition in each respective clone. Statistical significance determined using an ordinary two-way ANOVA and uncorrected Fisher's LSD with a single pooled variance. (n=3) (**D-E**) Average inter-passage proliferation rates (see methods) of individual knockout clones plotted against elapsed calendar days (**D**) or cumulative population doublings (**E**) fit with sigmoidal curves. (**F-G**) Linear (**F**) and sigmoidal (**G**) fits of knockout clone 2 average proliferation rate data. R^2 and Akaike information criterion (AICc) is shown, and residuals are plotted beneath the fit. Results of runs test for residual clustering are shown above the residual plot. (**H**) Schematic illustrating the creation of aspartate reared (AR) variants of knockout clones, which are cultured long-term in media supplemented with 20 mM aspartate, before aspartate is withdrawn and cells are evaluated for potential adaptation. (**I**) Average inter-passage proliferation rates (see methods) of aspartate reared (AR) clones 1-3 over 80-120 cumulative population doublings. (**J**) Absolute proliferation rates (mean +/- S.D.) of aspartate-reared (AR) SDHB knockout clones 1-3 with or without 20 mM aspartate supplementation. Statistical significance determined using an ordinary two-way ANOVA and uncorrected Fisher's LSD with a single pooled variance. (n=3) (**K**) Relative whole-cell aspartate levels (mean +/- S.D.) of early passage (EP), aspartate reared (AR), and late passage (LP) SDHB knockout clones 1-3. Levels are normalized to each corresponding EP clone. Statistical significance determined using an ordinary two-way ANOVA and Tukey's multiple comparisons test with a single pooled variance. (n=3) (**L**)Representative western blot showing levels of NDUFV2 and tubulin loading control in wild-type parental cells (WT), a previously adapted (PA) SDHB-KO clone that adapted by suppressing complex I [?] , and late passage (LP) SDHB knockout clones 1-3. Normalized NDUFV2 band densities are shown below each respective lane. Statistical significance determined using ordinary two-way ANOVAs and uncorrected Fisher's LSD with single pooled variance (**C,J**) or ordinary two-way ANOVA and Tukey's multiple comparisons test with a single pooled variance (**B,K**).

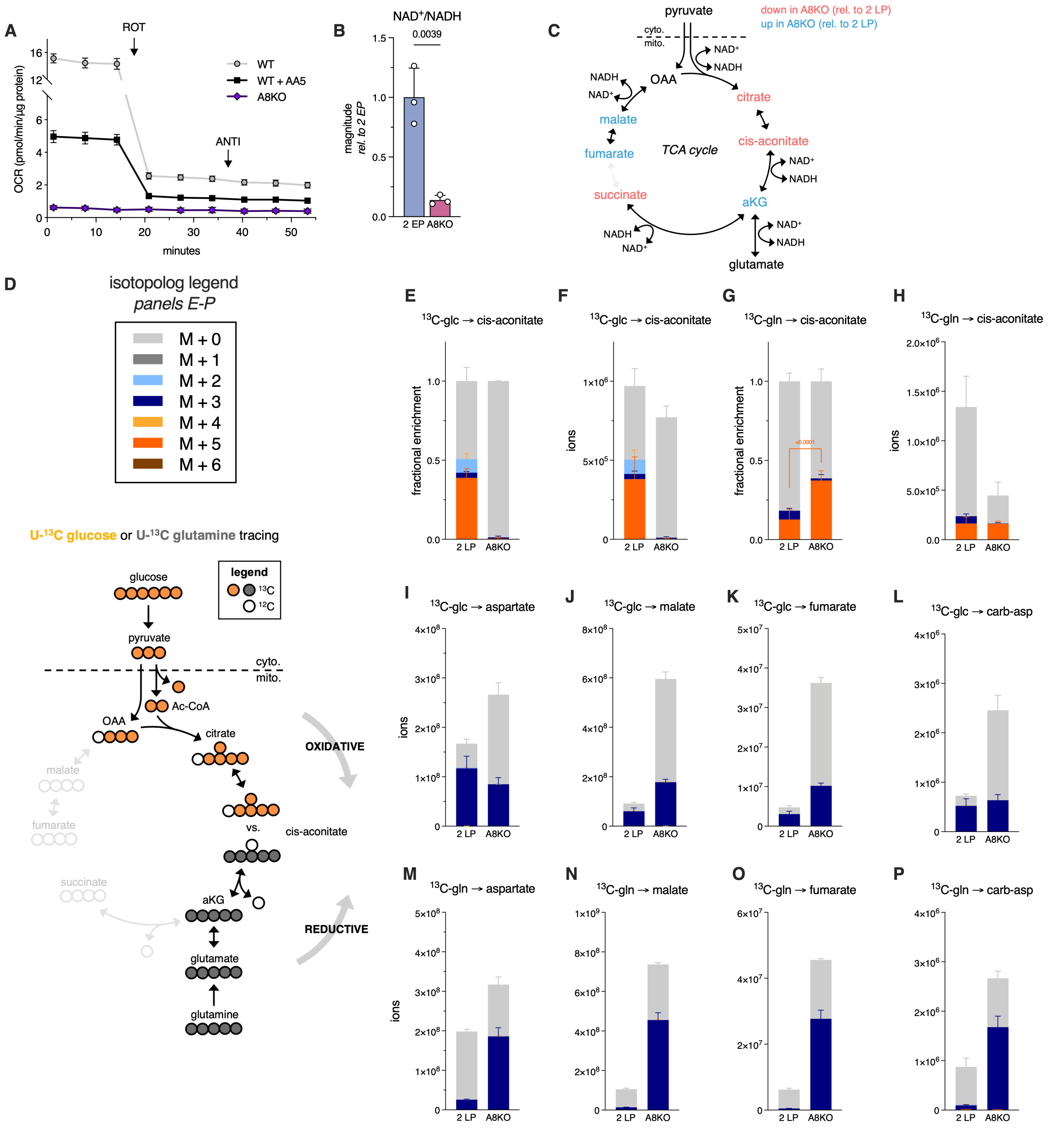

Fig. S2. Comparing adapted metabolic states of CI-high and CI-low SDH-deficient cells, related to Figure 2. (**A**) Normalized oxygen consumption rate (OCR) traces (mean +/- S.D.) for wild-type parental cells (WT), parental cells treated with 5 μM Atpenin A5 (WT + AA5), and early passage SDHB-KO NDUFA8-KO clone 2 (A8KO). Injections of rotenone (ROT) and antimycin (ANTI) are shown with arrows. (n=7-10) (**B**) Relative whole-cell NAD^+/NADH (mean +/- S.D.) in early passage SDHB KO clone 2 (2 EP) and A8KO 24 hours after media change. Levels are normalized to 2 EP. (n=3) (**C**) Schematic depicting TCA cycle metabolites which are more or less abundant in A8KO compared to late passage SDHB KO clone 2 (2 LP) (original data in Fig. 2E), highlighting redox-dependent interconversions. (**D**) Isotopolog legend for panels E-P and schematic illustrating U-^{13}C glucose/U-^{13}C glutamine tracing strategy to quantify oxidative vs. reductive TCA cycle metabolism in SDHB-KO cells. (**E-H**) Fractional (**E,G**) or absolute (**F,H**) isotopolog distributions (mean +/- S.D.) for cis-aconitate in 2 LP and A8KO following 24 hours of tracing in media containing U-^{13}C glucose (**E,F**) or U-^{13}C glutamine (**G,H**). In (**G**), p-value depicts the results of significance testing on the M+5 fraction. (n=3) (**I-P**) Absolute isotopolog distributions for the indicated metabolites (mean +/- S.D.) in late passage 2 LP and A8KO following 24 hours of tracing in media containing U-^{13}C glucose (**I-L**) or U-^{13}C glutamine (**M-P**). (n=3) Statistical significance determined using an unpaired t-test (**B**) or ordinary two-way ANOVA and Sidak's multiple comparisons test with a single pooled variance (**G**).

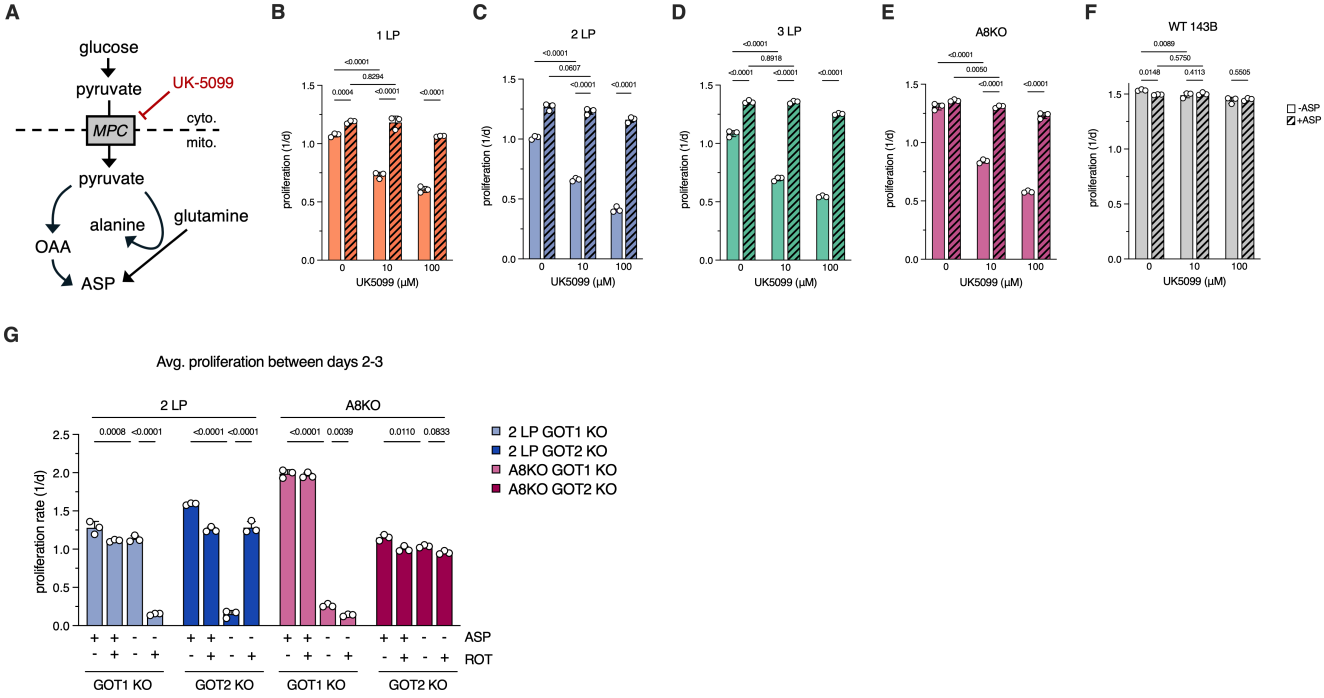

Fig. S3. Related to Figure 3. (**A**) Schematic illustrating the role of the mitochondrial pyruvate carrier (MPC) in alternative aspartate synthesis and its inhibition using UK-5099 (**B-F**) Proliferation rates (mean +/- S.D.) of late passage SDHB-KO clones 1-3 (1-3 LP) (**B-D**), A8KO (**E**), or wild-type 143B parental cells (143B) (**F**) following treatment with the indicated doses of UK-5099, with or without 20 mM aspartate supplementation. Statistical significance determined using an ordinary two-way ANOVA and uncorrected Fisher's LSD with a single pooled variance. (n=3) (**G**) Data from the same experiment as in Figure 3M, with proliferation rates (mean +/- S.D.) calculated between days 2-3 of the 3-day assay. Statistical significance determined using an ordinary two-way ANOVA and uncorrected Fisher's LSD with single pooled variance.

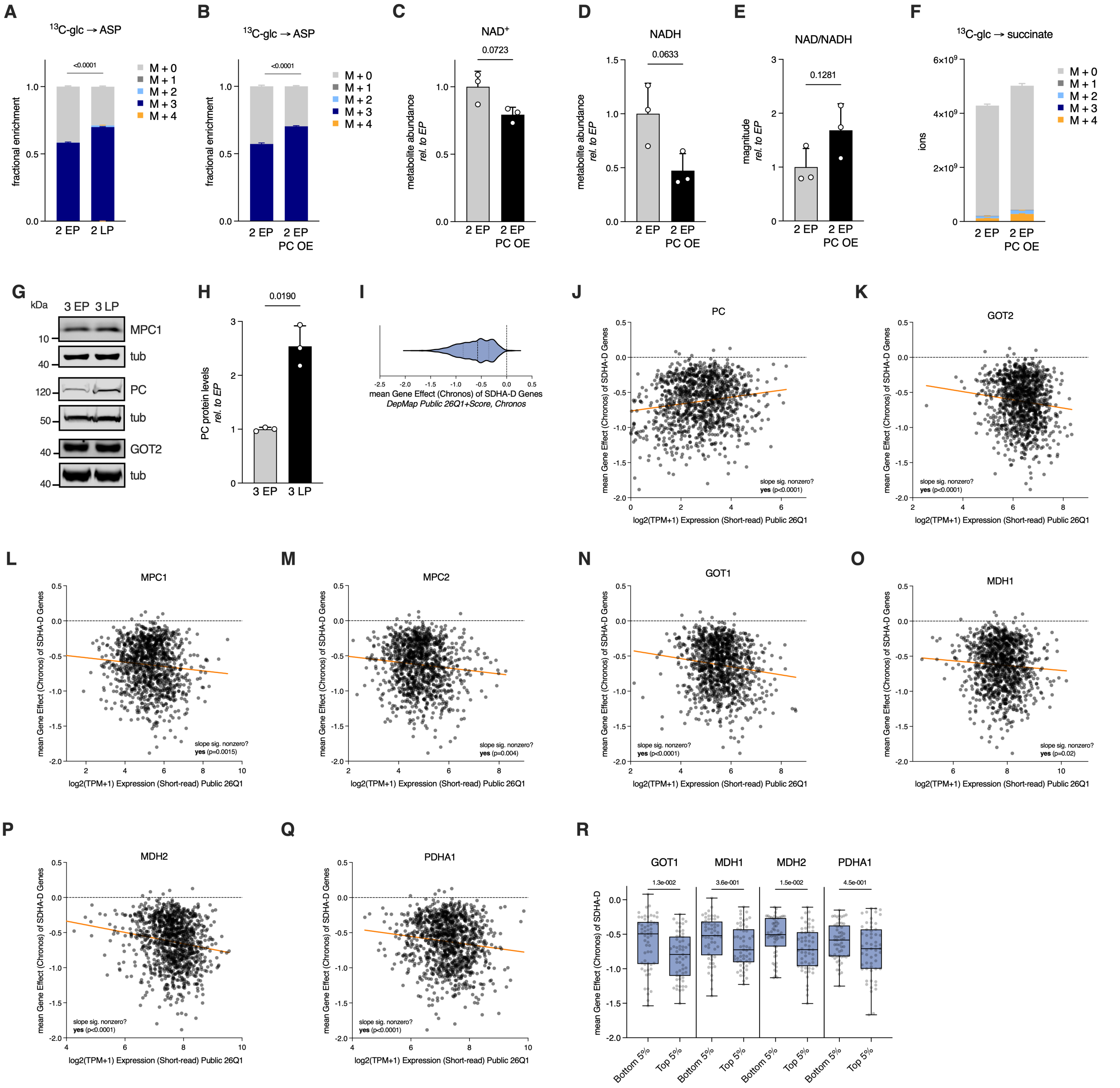

Fig. S4. Related to Figure 4. (**A**) Fractional isotopolog distributions (mean +/- S.D.) for aspartate in early/late passage (EP/LP) SDHB-KO clone 2 following 24 hours of tracing in media containing U-^{13}C glucose. (n=3) (**B**) Fractional isotopolog distributions (mean +/- S.D.) for aspartate in early passage SDHB-KO clone 2 (2 EP) and PC-overexpressing 2 EP following 24 hours of tracing in media containing U-^{13}C glucose. (n=3) (**C-E**) Relative NAD$^+$ (**C**), NADH (**D**), or NAD$^+$/NADH (**E**) levels (mean +/- S.D.) of 2 EP and 2 EP PC OE 24 hours after media change. Levels are normalized to 2 EP. (n=3) (**F**) Absolute isotopolog distributions (mean +/- S.D.) for succinate in 2 EP and 2 EP PC OE following 24 hours of tracing in media containing U-^{13}C glucose. (n=3) (**G**) Representative western blots showing levels of MPC1, PC, GOT2, and tubulin loading control in early passage (EP) and late passage (LP) SDHB KO clone 3. (**H**) Quantification of PC protein levels from 3 EP/LP using western blotting. (n=3) (**I**) Mean SDHA-D gene effect scores for all cell lines in DepMap 26Q1 (**J-Q**) Mean SDHA-D gene effect scores for cell lines in DepMap 26Q1 plotted against relative expression of PC (**J**), GOT2 (**K**), MPC1 (**L**), MPC2 (**M**), GOT1 (**N**), MDH1 (**O**), MDH2 (**P**), and PDHA1 (**Q**) fitted with linear regressions (n=1140 cell lines) (**R**) Mean SDHA-D DepMap gene effect scores for the top and bottom 5% of expressors of the indicated metabolic genes. (n=57) Statistical significance determined using Welch's t-test (**C-E, H**), or ordinary one-way (**R**) or two-way (**A-B**)ANOVAs with Sidak's multiple comparisons test with a single pooled variance. Whether the slope of the regression lines were significantly nonzero was determined using Prism.

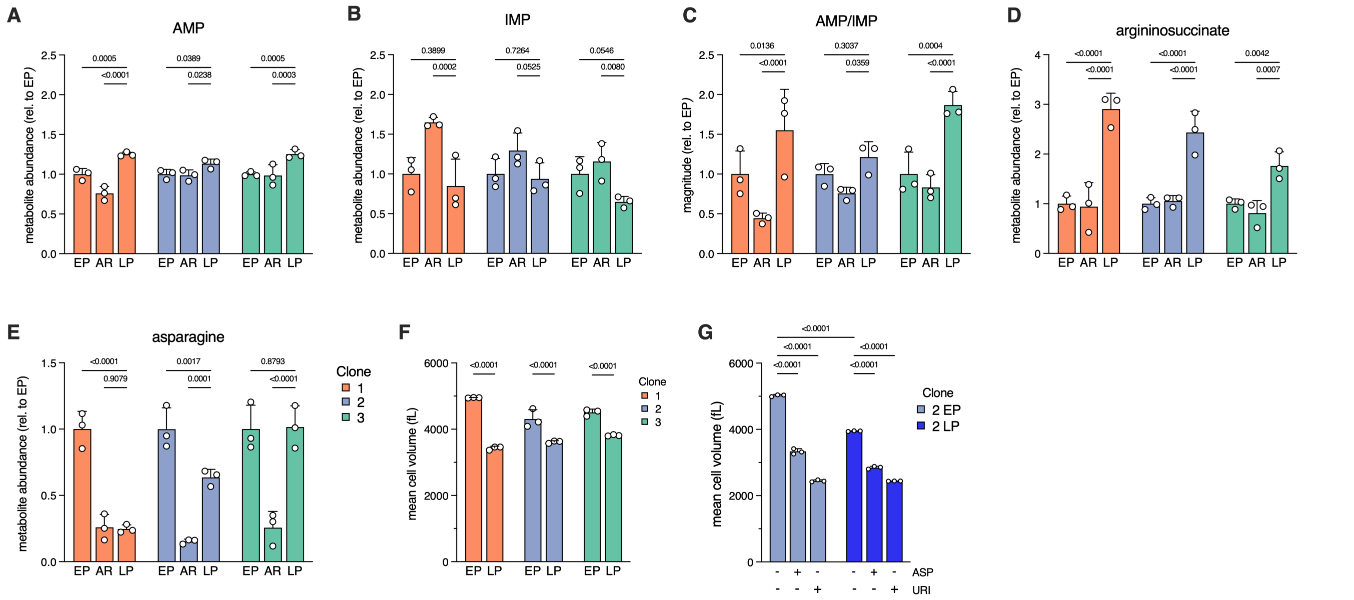

Fig. S5. Related to Figure 5. (**A-E**) Relative levels of adenosine monophosphate (AMP) (**A**), inosine monophosphate (IMP) (**B**), AMP/IMP (**C**), argininosuccinate (**D**), or asparagine (**E**) (mean +/- S.D.) in early passage (EP), aspartate reared (AR), and late passage (LP) SDHB knockout clones 1-3. Levels are normalized to each corresponding EP clone. (n=3) (**F**) Mean per-cell volumes of EP/LP SDHB KO clones 1-3. (n=3) (**G**) Mean per-cell volume of EP/LP SDHB KO clone 2 supplemented with 20 mM aspartate (ASP) or 500 μM uridine (URI). (n=3) Statistical significance determined using ordinary two-way ANOVAs and uncorrected Fisher's LSD with single pooled variance (**A-F**) or ordinary two-way ANOVAs and Tukey's multiple comparisons test with single pooled variance (**G**).

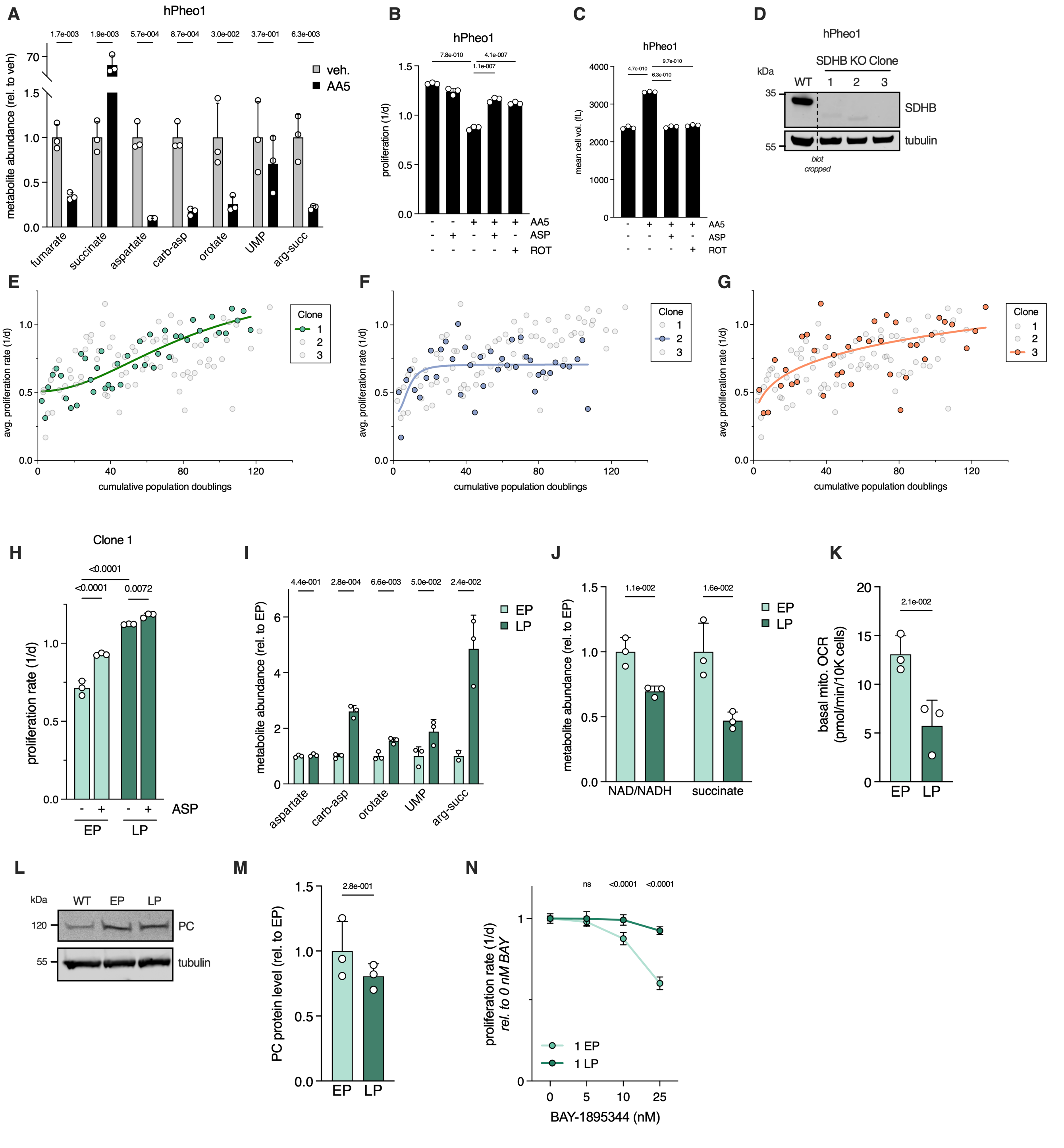

Fig. S6. Adaptation in SDH-deficient human pheochromocytoma cells. (**A**) Relative abundance (mean +/- S.D.) of the indicated metabolites in hPheo1 cells treated with 5 μM AA5 or vehicle control for 24 hours. Metabolite abundances are normalized to vehicle-treated controls. (n=3). (**B**) Proliferation rates (mean +/- S.D.) of hPheo1 cells treated with the indicated combinations of 5 μM AA5, 20 mM aspartate (ASP), and 100 nM rotenone (ROT). (n=3). (**C**) Mean per-cell volume (mean +/- S.D.) of hPheo1 cells with the indicated combinations of 5 μM AA5, 20 mM aspartate (ASP), and 100 nM rotenone (ROT). (n=3). (**D**) Western blot showing expression of SDHB and tubulin loading control in wild-type (WT) hPheo1 cells and three hPheo1 SDHB knockout clones. (**E-G**) Average inter-passage proliferation rates (see methods) of hPheo1 SDHB KO clones 1-3 over 120 cumulative population doublings, with overlaid sigmoidal curve fit. (**H**) Proliferation rates (mean +/- S.D.) of early passage (EP) and late passage (LP) hPheo1 SDHB KO clone 1 with or without 20 mM aspartate (ASP) supplementation. (n=3). (**I**) Relative abundance (mean +/- S.D.) of the indicated metabolites in EP/LP hPheo1 SDHB KO clone 1. Metabolite abundances are normalized to EP. (n=3). (**J**) Relative magnitude (mean +/- S.D.) of NAD^+/NADH and succinate abundance in EP/LP hPheo1 SDHB KO clone 1. Metabolite abundances are normalized to EP. (n=3). (**K**) Basal mitochondrial oxygen consumption rates (mito. OCR) (mean +/- S.D.) of EP/LP hPheo1 SDHB KO clone 1. (n=3). (**L**) Western blot showing expression of PC and tubulin loading control in wild-type (WT) hPheo1 cells and EP/LP hPheo1 SDHB KO clone 1 (**M**) PC protein levels (mean +/- S.D.) in EP/LP hPheo1 SDHB KO clone 1 determined using western blotting of replicate samples and densitometry. (n=3). (**N**) Proliferation rates (mean +/- S.D.) of EP/LP hPheo1 SDHB knockout clone 1 in the indicated doses of BAY-1895344. Proliferation rates are normalized to the 0 nM BAY dose in each condition. P-values shown correspond to statistical testing comparing the relative proliferation rate of EP vs. LP at the indicated dose of BAY. (n=3) Statistical significance determined using multiple unpaired t-tests (**A,I-J**), ordinary one-way (**B-C**) or two-way (**H**) ANOVAs and Tukey's multiple comparisons test with single pooled variance (**B-C**), ordinary two-way ANOVAs and an uncorrected Fisher's LSD with a single pooled variance (**N**), or Welch's t-tests (**K,M**).

Clone	Bottom (doublings/day)	Top (doublings/day)	Span (doublings/day)	IC50 (doublings)	R^2
1	0.45	0.78	0.32	33.75	0.50
2	0.44	0.82	0.38	39.87	0.62
3	0.42	0.89	0.47	41.36	0.74
4	0.37	0.83	0.45	33.57	0.77
5	0.20	1.09	0.88	46.54	0.73
avg.	0.37	0.882	0.5	39.01	

Supplemental Table 1. Table of fitted curve parameters for each knockout clone (plotted in Figure S1E) and the average of all five clones, including bottom (doublings/day), top (doublings/day), span (top-bottom, doublings/day), 'IC50' (the number of cumulative population doublings needed to achieve half of the 'top' proliferation rate), and R^2.

Key Resources Table

Resource/Reagent	Manufacturer	Catalog Number	Sequence (5'-3') if applicable	Dose/dilution used
SDHB sgRNA 1	Synthego	N/A	UCGCCCUCUCCUUGAGGCG	See Methods
SDHB sgRNA 2	Synthego	N/A	AGAAAUUUGCCAUCUAUCGA	See Methods
NDUFA8 sgRNA 1	Synthego	N/A	CACAUUGAGCUCCAUAGUGA	See Methods
NDUFA8 sgRNA 2	Synthego	N/A	AAAGCUGCGGCCCAUCACUA	See Methods
NDUFA8 sgRNA 3	Synthego	N/A	UUCUGCUGUGCUUAAAGCUG	See Methods
MPC1 sgRNA 1	Synthego	N/A	UUGCCUACAAGGUACAGCCU	See Methods
MPC1 sgRNA 2	Synthego	N/A	GGGCUACUUCAUUUGUUGCG	See Methods
MPC1 sgRNA 3	Synthego	N/A	AUGUCAAAGAAUAGCAACAG	See Methods
PC sgRNA 1	Synthego	N/A	GCAGGCCCGGAACACACGGA	See Methods
PC sgRNA 2	Synthego	N/A	GCUGGAGGAGAAUUACACCC	See Methods
PC sgRNA 3	Synthego	N/A	ACACCGGCCGCAUUGAGGU	See Methods
GOT1 sgRNA 1	Synthego	N/A	CAGUCAUCCGUGCGAUAUGC	See Methods
GOT1 sgRNA 2	Synthego	N/A	GCACGGAUGACUGCCAUCCC	See Methods
GOT1 sgRNA 3	Synthego	N/A	CGAUCUUCUCCAUCUGGGAA	See Methods
GOT2 sgRNA 1	Synthego	N/A	UUUCUCAUUUCAGCUCCUGG	See Methods
GOT2 sgRNA 2	Synthego	N/A	CGGACGCUAGGCAGAACGUA	See Methods
GOT2 sgRNA 3	Synthego	N/A	UCCUUCCACUGUUCCGGACG	See Methods
Aspartate	Sigma	A7219	N/A	20 mM
Uridine	Sigma	U3003	N/A	500 μM
UK-5099	Cayman Chemical	16980	N/A	Varies, see figure
Rotenone	Sigma	R8875	N/A	50-100 nM
Antimycin	Sigma	A8674	N/A	500 nM
Anti-SDHB	Atlas Antibodies	HPA002868	N/A	1:1000
Anti-tubulin	Sigma	T6199	N/A	1:3000
Anti-V5	Cell Signaling	13202S	N/A	1:2000
Anti-NDUFV2	Proteintech	15301-1-AP	N/A	1:1000
Anti-NDUFA8	Atlas Antibodies	HPA041510	N/A	1:1000
Anti-MPC1	Cell Signaling	14462S	N/A	1:1000
Anti-PC	Proteintech	66615-1	N/A	1:1000
Anti-GOT2	Proteintech	14800-1-AP	N/A	1:1000
Anti-GOT1	Cell Signaling	34423S	N/A	1:1000
pLX304_PC	DNASU Plasmid Repository	HsCD00436386	N/A	N/A
pLenti6.3-V5-DEST_SDHB	DNASU Plasmid Repository	HsCD00954622	N/A	N/A
pLenti6.3-V5-DEST_MPC1	DNASU Plasmid Repository	HsCD00942383	N/A	N/A
BAY-1895344	Selleckchem	S8666	N/A	Varies, see figure
