# Supplementary material for: Adaptive plasticity of aspartate metabolism in succinate dehydrogenase-deficient cancer cells
Source: bioRxiv. 2026 May 21:2026.05.18.726122. Preprint. [Version 1] doi: 10.64898/2026.05.18.726122 (PMC13228284; doi:10.64898/2026.05.18.726122)
Supplement: Supplementary file 2 [file media-1.pdf]

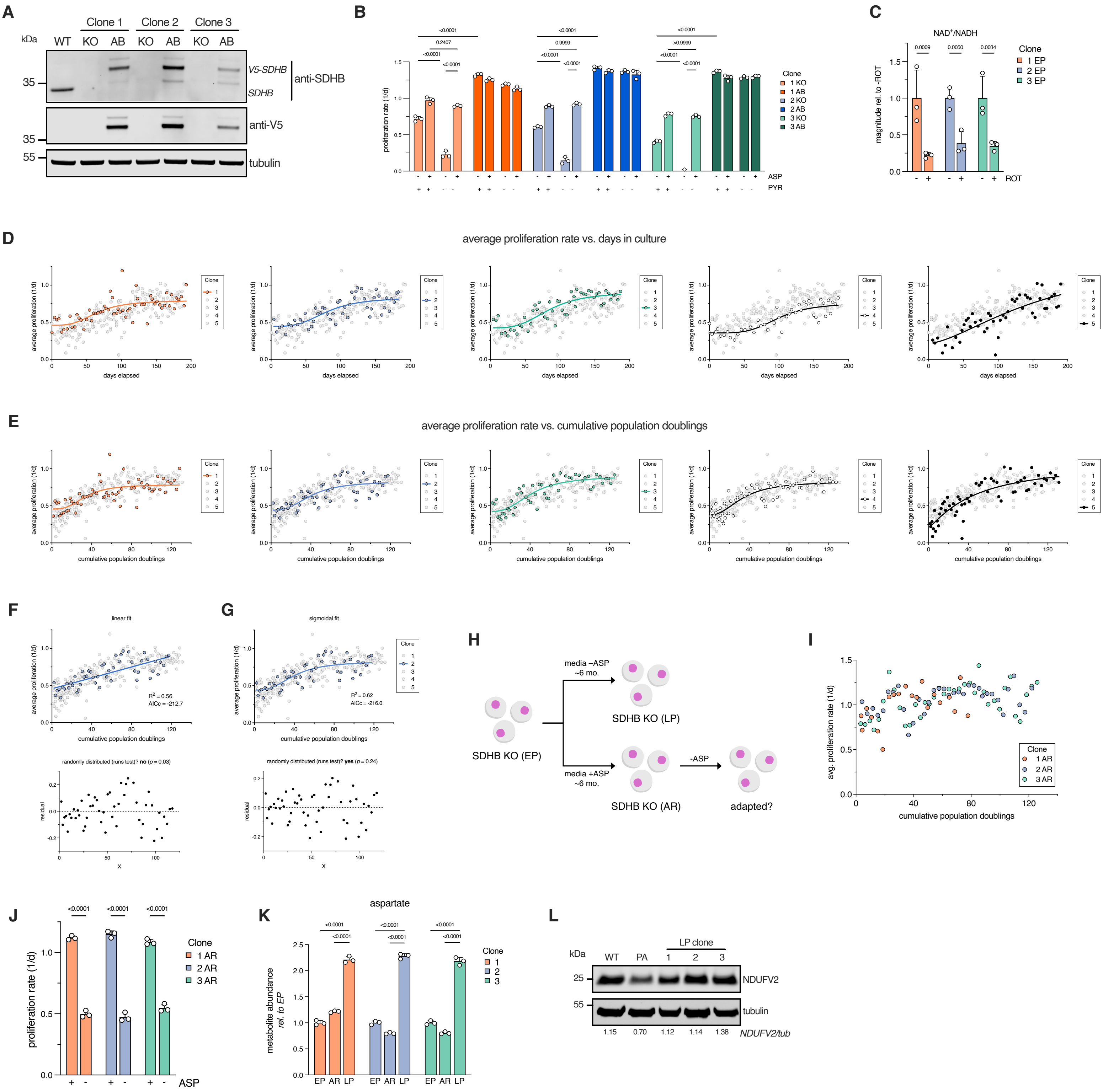

A
kDa
Clone 1
Clone 2
Clone 3
WT KO AB KO AB KO AB
35
V5-SDHB
SDHB
anti-SDHB
35
anti-V5
55
tubulin
B
proliferation rate (1/d)
<0.0001
0.2407
<0.0001
<0.0001
<0.0001
0.9999
<0.0001
<0.0001
<0.0001
>0.9999
<0.0001
<0.0001
ASP
PYR
Clone
1 KO
1 AB
2 KO
2 AB
3 KO
3 AB
C
NAD+/NADH
magnitude rel. to -ROT
0.0009
0.0050
0.0034
ROT
Clone
1 EP
2 EP
3 EP
D
average proliferation rate vs. days in culture
average proliferation (1/d)
days elapsed
Clone
1
2
3
4
5
E
average proliferation rate vs. cumulative population doublings
average proliferation (1/d)
cumulative population doublings
F
linear fit
R2 = 0.56
AICc = -212.7
randomly distributed (runs test)? no (p = 0.03)
residual
x
G
sigmoidal fit
R2 = 0.62
AICc = -216.0
randomly distributed (runs test)? yes (p = 0.24)
H
SDHB KO (EP)
media –ASP
~6 mo.
SDHB KO (LP)
media +ASP
~6 mo.
SDHB KO (AR)
-ASP
adapted?
I
avg. proliferation rate (1/d)
cumulative population doublings
Clone
1 AR
2 AR
3 AR
J
proliferation rate (1/d)
<0.0001
<0.0001
<0.0001
ASP
Clone
1 AR
2 AR
3 AR
K
aspartate
metabolite abundance
rel. to EP
<0.0001
<0.0001
<0.0001
<0.0001
<0.0001
<0.0001
EP AR LP
Clone
1
2
3
L
kDa
WT PA
LP clone
1 2 3
25
NDUFV2
55
tubulin
1.15 0.70 1.12 1.14 1.38
NDUFV2/tub
