# Supplementary material for: Adaptive plasticity of aspartate metabolism in succinate dehydrogenase-deficient cancer cells
Source: bioRxiv. 2026 May 21:2026.05.18.726122. Preprint. [Version 1] doi: 10.64898/2026.05.18.726122 (PMC13228284; doi:10.64898/2026.05.18.726122)
Supplement: Supplementary file 3 [file media-2.pdf]

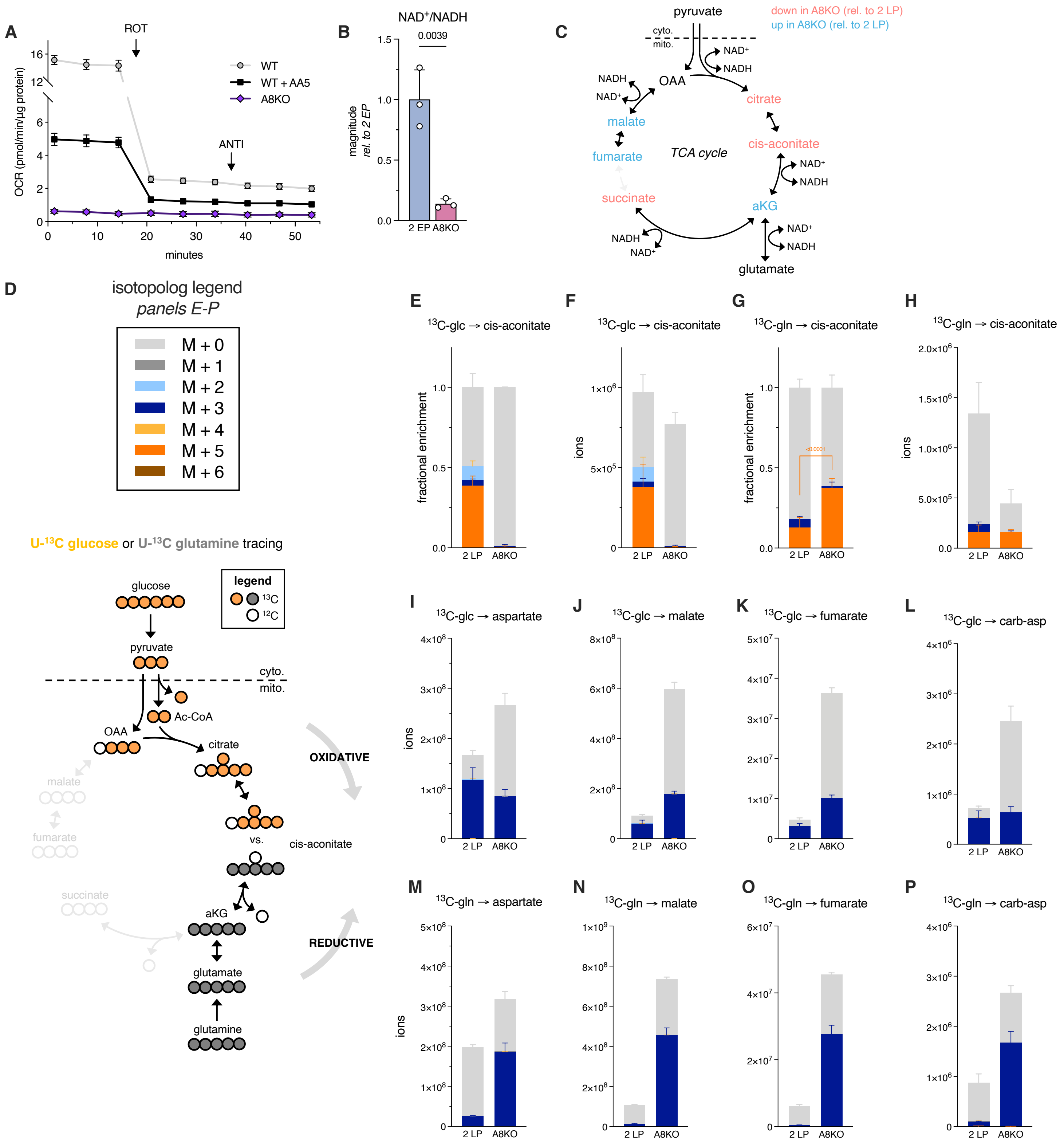
A
ROT
ANTI
OCR (pmol/min/µg protein)
minutes
WT
WT + AA5
A8KO
B
NAD+/NADH
0.0039
magnitude
rel. to 2 EP
2 EP
A8KO
C
pyruvate
cyto.
mito.
down in A8KO (rel. to 2 LP)
up in A8KO (rel. to 2 LP)
NAD+
NADH
OAA
citrate
malate
fumarate
TCA cycle
cis-aconitate
succinate
aKG
glutamate
D
isotopolog legend
panels E-P
M + 0
M + 1
M + 2
M + 3
M + 4
M + 5
M + 6
U-13C glucose or U-13C glutamine tracing
glucose
legend
13C
12C
pyruvate
Ac-CoA
OAA
citrate
malate
fumarate
OXIDATIVE
vs.
cis-aconitate
succinate
aKG
REDUCTIVE
glutamate
glutamine
E
13C-glc → cis-aconitate
fractional enrichment
F
13C-glc → cis-aconitate
ions
G
13C-gln → cis-aconitate
fractional enrichment
<0.0001
H
13C-gln → cis-aconitate
ions
I
13C-glc → aspartate
J
13C-glc → malate
K
13C-glc → fumarate
L
13C-glc → carb-asp
M
13C-gln → aspartate
N
13C-gln → malate
O
13C-gln → fumarate
P
13C-gln → carb-asp
2 LP
A8KO
