# Supplementary material for: Adaptive plasticity of aspartate metabolism in succinate dehydrogenase-deficient cancer cells
Source: bioRxiv. 2026 May 21:2026.05.18.726122. Preprint. [Version 1] doi: 10.64898/2026.05.18.726122 (PMC13228284; doi:10.64898/2026.05.18.726122)
Supplement: Supplementary file 4 [file media-3.pdf]

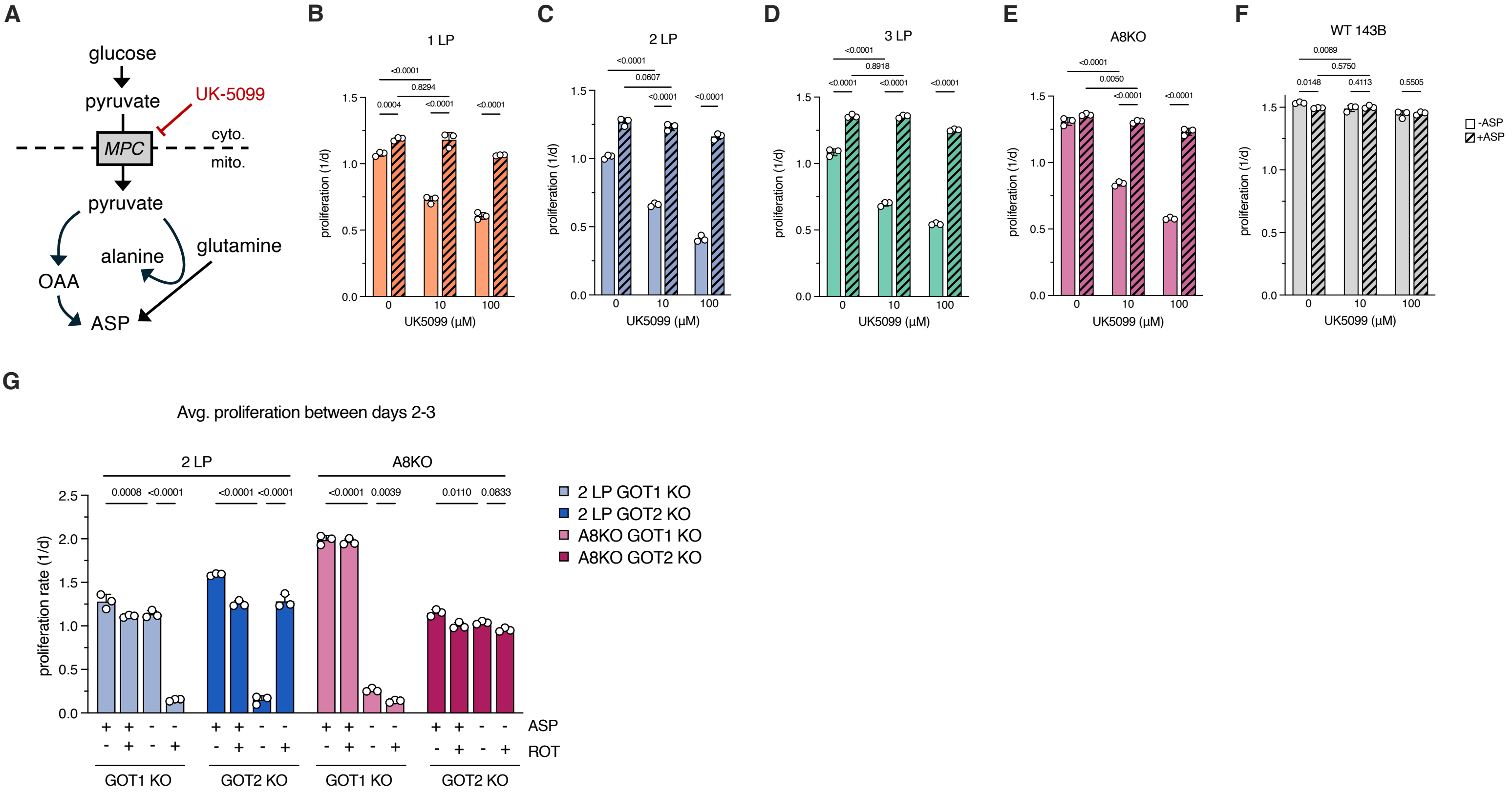

A
glucose
pyruvate
UK-5099
MPC
cyto.
mito.
pyruvate
alanine
glutamine
OAA
ASP
B
1 LP
C
2 LP
D
3 LP
E
A8KO
F
WT 143B
-ASP
+ASP
proliferation (1/d)
UK5099 (μM)
0
10
100
G
Avg. proliferation between days 2-3
2 LP
A8KO
proliferation rate (1/d)
2 LP GOT1 KO
2 LP GOT2 KO
A8KO GOT1 KO
A8KO GOT2 KO
ASP
ROT
GOT1 KO
GOT2 KO
GOT1 KO
GOT2 KO
