# Supplementary material for: Adaptive plasticity of aspartate metabolism in succinate dehydrogenase-deficient cancer cells
Source: bioRxiv. 2026 May 21:2026.05.18.726122. Preprint. [Version 1] doi: 10.64898/2026.05.18.726122 (PMC13228284; doi:10.64898/2026.05.18.726122)
Supplement: Supplementary file 5 [file media-4.pdf]

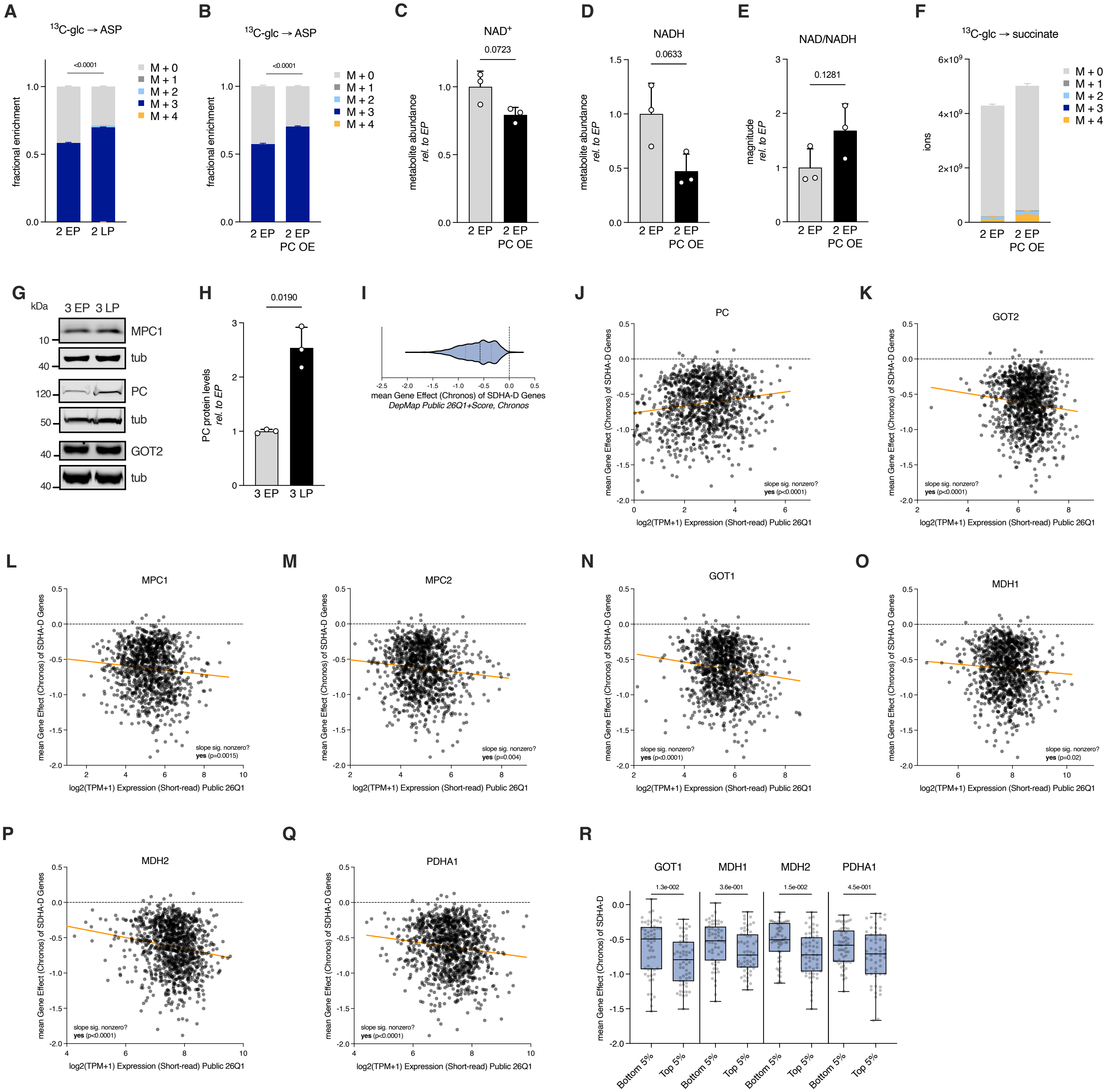

A
13C-glc → ASP
<0.0001
fractional enrichment
2 EP
2 LP
M + 0
M + 1
M + 2
M + 3
M + 4
B
13C-glc → ASP
<0.0001
fractional enrichment
2 EP
2 EP PC OE
C
NAD+
0.0723
metabolite abundance rel. to EP
2 EP
2 EP PC OE
D
NADH
0.0633
metabolite abundance rel. to EP
E
NAD/NADH
0.1281
magnitude rel. to EP
F
13C-glc → succinate
ions
G
kDa
3 EP
3 LP
MPC1
tub
PC
GOT2
H
0.0190
PC protein levels rel. to EP
3 EP
3 LP
I
mean Gene Effect (Chronos) of SDHA-D Genes
DepMap Public 26Q1+Score, Chronos
J
PC
K
GOT2
L
MPC1
M
MPC2
N
GOT1
O
MDH1
P
MDH2
Q
PDHA1
mean Gene Effect (Chronos) of SDHA-D Genes
log2(TPM+1) Expression (Short-read) Public 26Q1
slope sig. nonzero?
yes (p<0.0001)
yes (p=0.0015)
yes (p=0.004)
yes (p=0.02)
R
GOT1
MDH1
MDH2
PDHA1
1.3e-002
3.6e-001
1.5e-002
4.5e-001
mean Gene Effect (Chronos) of SDHA-D
Bottom 5%
Top 5%
