# Supplementary material for: Adaptive plasticity of aspartate metabolism in succinate dehydrogenase-deficient cancer cells
Source: bioRxiv. 2026 May 21:2026.05.18.726122. Preprint. [Version 1] doi: 10.64898/2026.05.18.726122 (PMC13228284; doi:10.64898/2026.05.18.726122)
Supplement: Supplementary file 6 [file media-5.pdf]

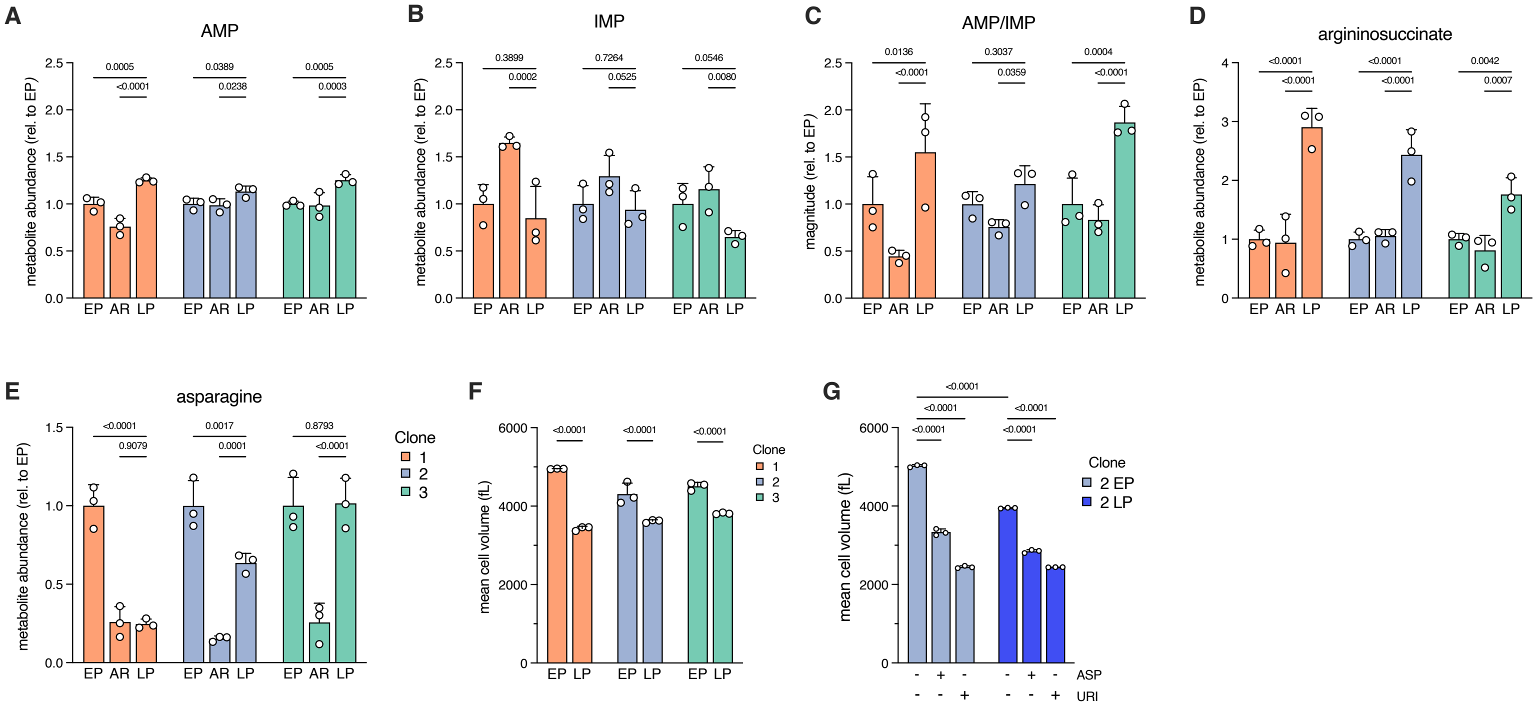

A
AMP
metabolite abundance (rel. to EP)
0.0005
<0.0001
0.0389
0.0238
0.0005
0.0003
EP AR LP EP AR LP EP AR LP
B
IMP
0.3899
0.0002
0.7264
0.0525
0.0546
0.0080
C
AMP/IMP
magnitude (rel. to EP)
0.0136
<0.0001
0.3037
0.0359
0.0004
<0.0001
D
argininosuccinate
<0.0001
<0.0001
<0.0001
<0.0001
0.0042
0.0007
E
asparagine
<0.0001
0.9079
0.0017
0.0001
0.8793
<0.0001
Clone
1
2
3
F
mean cell volume (fL)
<0.0001
<0.0001
<0.0001
EP LP EP LP EP LP
G
<0.0001
<0.0001
<0.0001
<0.0001
<0.0001
2 EP
2 LP
ASP
URI
