# Supplementary material for: Adaptive plasticity of aspartate metabolism in succinate dehydrogenase-deficient cancer cells
Source: bioRxiv. 2026 May 21:2026.05.18.726122. Preprint. [Version 1] doi: 10.64898/2026.05.18.726122 (PMC13228284; doi:10.64898/2026.05.18.726122)
Supplement: Supplementary file 7 [file media-6.pdf]

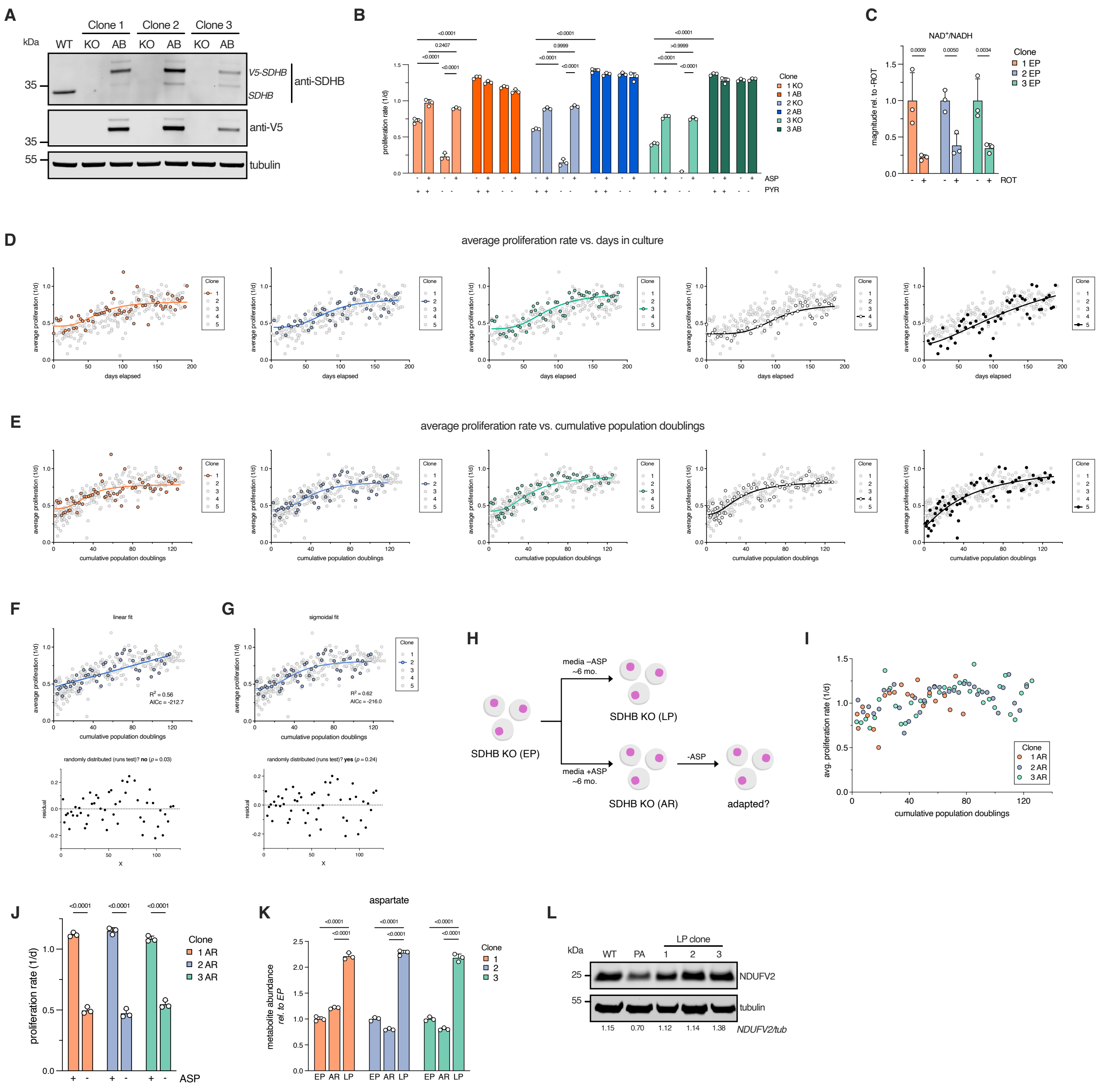

Figure S1. related to Figure 1.

(A) Representative western blot showing levels of SDHB, V5-tagged SDHB, and tubulin loading control in wild-type parental cells (WT), early passage (EP), and addback (AB) SDHB knockout clones 1-3.

(B) Absolute proliferation rates (mean +/- S.D.) of early passage (EP) and addback (AB) clones 1-3 in DMEM with all combinations of 1 mM pyruvate and 20 mM aspartate supplementation. Statistical significance determined using an ordinary two-way ANOVA and Tukey's multiple comparisons test with a single pooled variance. Data for KO clones in pyruvate-containing media are the same as plotted in Figure 1H. (n=3)

(C) Relative whole-cell NAD^+/NADH (mean +/- S.D.), measured using LCMS, in SDHB-KO clones 1-3 after 6 hours of treatment with vehicle control or 50 nM rotenone (ROT). Values

are normalized to the vehicle-treated condition in each respective clone. Statistical significance determined using an ordinary two-way ANOVA and uncorrected Fisher's LSD with a single pooled variance. (n=3)

(D) Average inter-passage proliferation rates (see methods) of individual knockout clones plotted against elapsed calendar days fit with sigmoidal curves.

(E) Average inter-passage proliferation rates (see methods) of individual knockout clones plotted against cumulative population doublings fit with sigmoidal curves.

(F) Linear fit of knockout clone 2 average proliferation rate data. R^2 and Akaike information criterion (AICc) is shown, and residuals are plotted beneath the fit. Results of runs test for residual clustering are shown above the residual plot.

(G) Sigmoidal fit of knockout clone 2 average proliferation data, as compared to (F)

(H) Schematic illustrating the creation of aspartate reared (AR) variants of knockout clones, which are cultured long-term in media supplemented with 20 mM aspartate, before aspartate is withdrawn and cells are evaluated for potential adaptation.

(I) Average inter-passage proliferation rates (see methods) of aspartate reared (AR) clones 1-3 over ~80-120 cumulative population doublings.

(J) Absolute proliferation rates (mean +/- S.D.) of aspartate-reared (AR) SDHB knockout clones 1-3 with or without 20 mM aspartate supplementation. Statistical significance determined using an ordinary two-way ANOVA and uncorrected Fisher's LSD with a single pooled variance. (n=3)

(K) Relative whole-cell aspartate levels (mean +/- S.D.) of early passage (EP), aspartate reared (AR), and late passage (LP) SDHB knockout clones 1-3. Levels are normalized to each corresponding EP clone. Statistical significance determined using an ordinary two-way ANOVA and Tukey's multiple comparisons test with a single pooled variance. (n=3)

(L) Representative western blot showing levels of NDUFV2 and tubulin loading control in wild-type parental cells (WT), a previously adapted (PA) SDHB-KO clone that adapted by suppressing complex I, and late passage (LP) SDHB knockout clones 1-3. Normalized NDUFV2 band densities are shown below each respective lane.

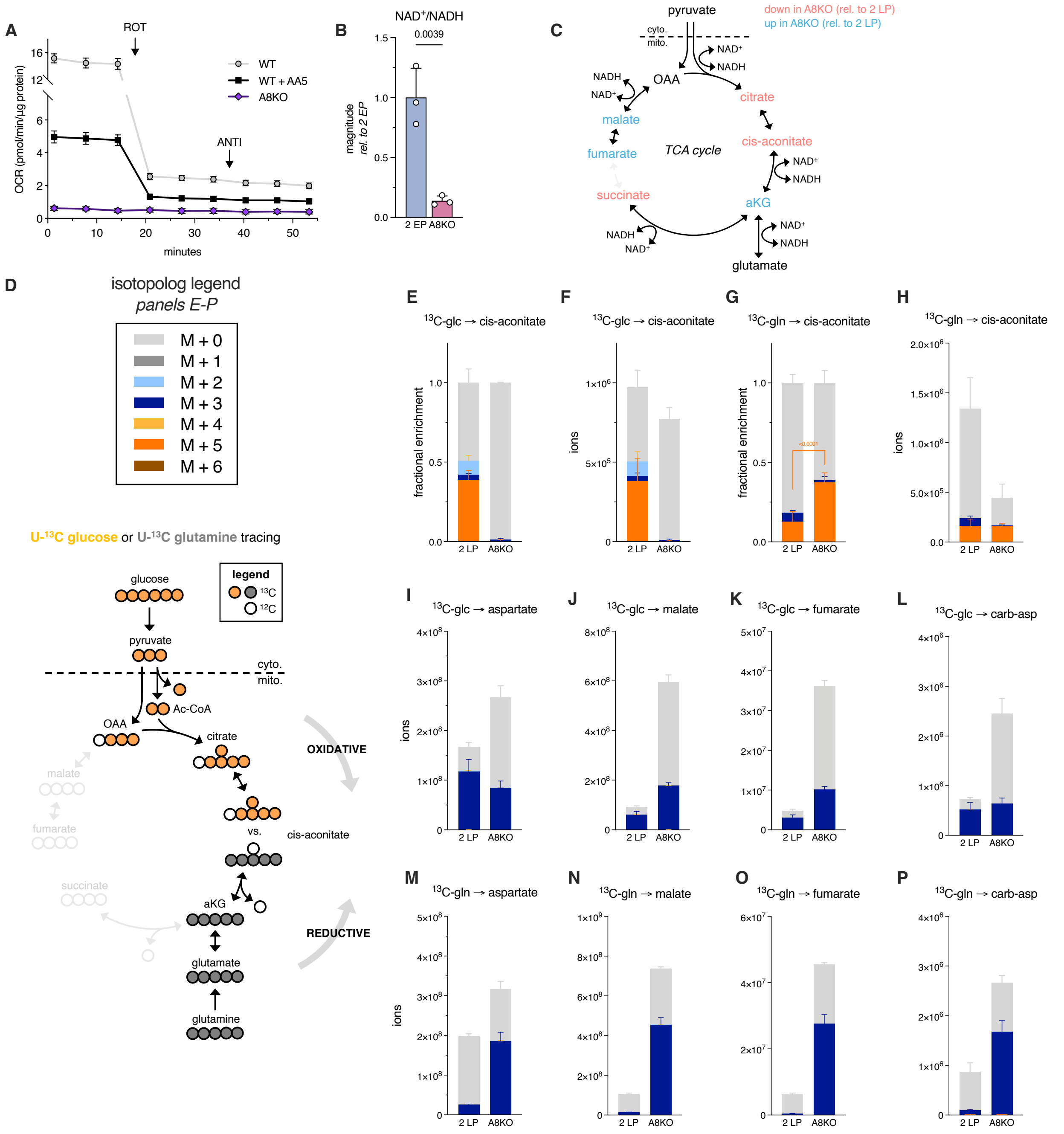

Figure S2. Comparing adapted metabolic states of CI-high and CI-low SDH-deficient cells, related to Figure 2

(A) normalized oxygen consumption rate (OCR) traces (mean +/- S.D.) for wild-type parental cells (WT), parental cells treated with 5 uM Atpenin A5 (WT + AA5), and early passage SDHB-KO NDUFA8-KO clone 2 (A8KO). Injections of rotenone (ROT) and antimycin (ANTI) are shown with arrows. (n=7-10)

(B) Relative whole-cell NAD$^+$/NADH (mean +/- S.D.) in early passage SDHB KO clone 2 (2 EP) and A8KO 24 hours after media change. Levels are normalized to 2 EP. Statistical significance determined using an unpaired t-test. (n=3)

(C) Schematic depicting TCA cycle metabolites which are more or less abundant in A8KO compared to late passage SDHB KO clone 2 (2 LP) (original data in Fig. 2E), highlighting redox-dependent interconversions.

(D) Isotopolog legend for panels E-P and schematic illustrating U-^{13}C glucose/U-^{13}C glutamine tracing strategy to quantify oxidative vs. reductive TCA cycle metabolism in SDHB-KO cells

(E-H) fractional (E, G) or absolute (F, H) isotopolog distributions (mean +/- S.D.) for cis-aconitate in 2 LP and A8KO following 24 hours of tracing in media containing U-^{13}C glucose (E, F) or U-^{13}C glutamine (G, H). In (G), p-value depicts the results of significance testing on the M+5 fraction using an ordinary two-way ANOVA with Sidak's multiple comparisons test and a single pooled variance. (n=3)

(I-P) absolute isotopolog distributions for the indicated metabolites (mean +/- S.D.) in late passage 2 LP and A8KO following 24 hours of tracing in media containing U-^{13}C glucose (I-L) or U-^{13}C glutamine (M-P). (n=3)

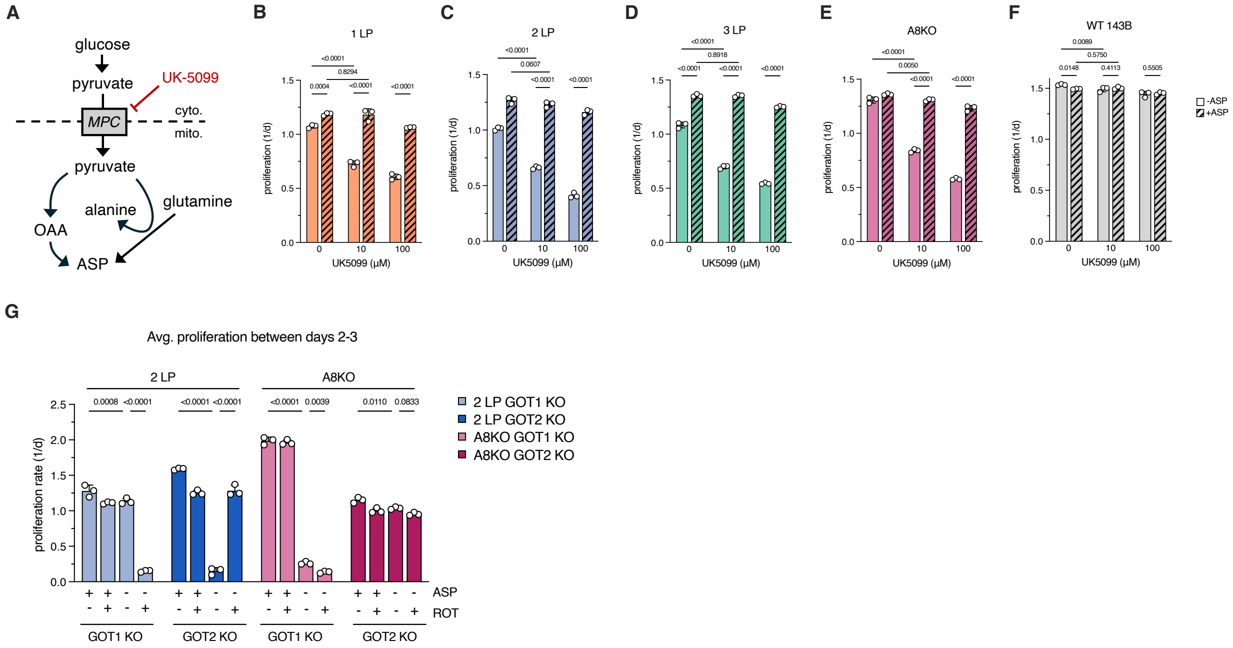

Figure S3. related to Figure 3

(A) Schematic illustrating the role of the mitochondrial pyruvate carrier (MPC) in alternative aspartate synthesis and its inhibition using UK-5099

(B) Proliferation rates (mean +/- S.D.) of late passage SDHB-KO clone 1 (1 LP) following treatment with the indicated doses of UK-5099, with or without 20 mM aspartate supplementation. Statistical significance determined using an ordinary two-way ANOVA and uncorrected Fisher's LSD with a single pooled variance. (n=3)

(C) Identical setup as in (B), with late passage SDHB-KO clone 2 (2 LP) (n=3)

(D) Identical setup as in (B), with late passage SDHB-KO clone 3 (3 LP) (n=3)

(E) Identical setup as in (B), with NDUFA8-KO early passage SDHB KO clone 2 (A8KO) (n=3)

(F) Identical setup as in (B), with wild-type 143B parental cells (n=3)

(G) Data from the same experiment as in Figure 3M, with proliferation rates (mean +/- S.D.) calculated between days 2-3 of the 3-day assay.

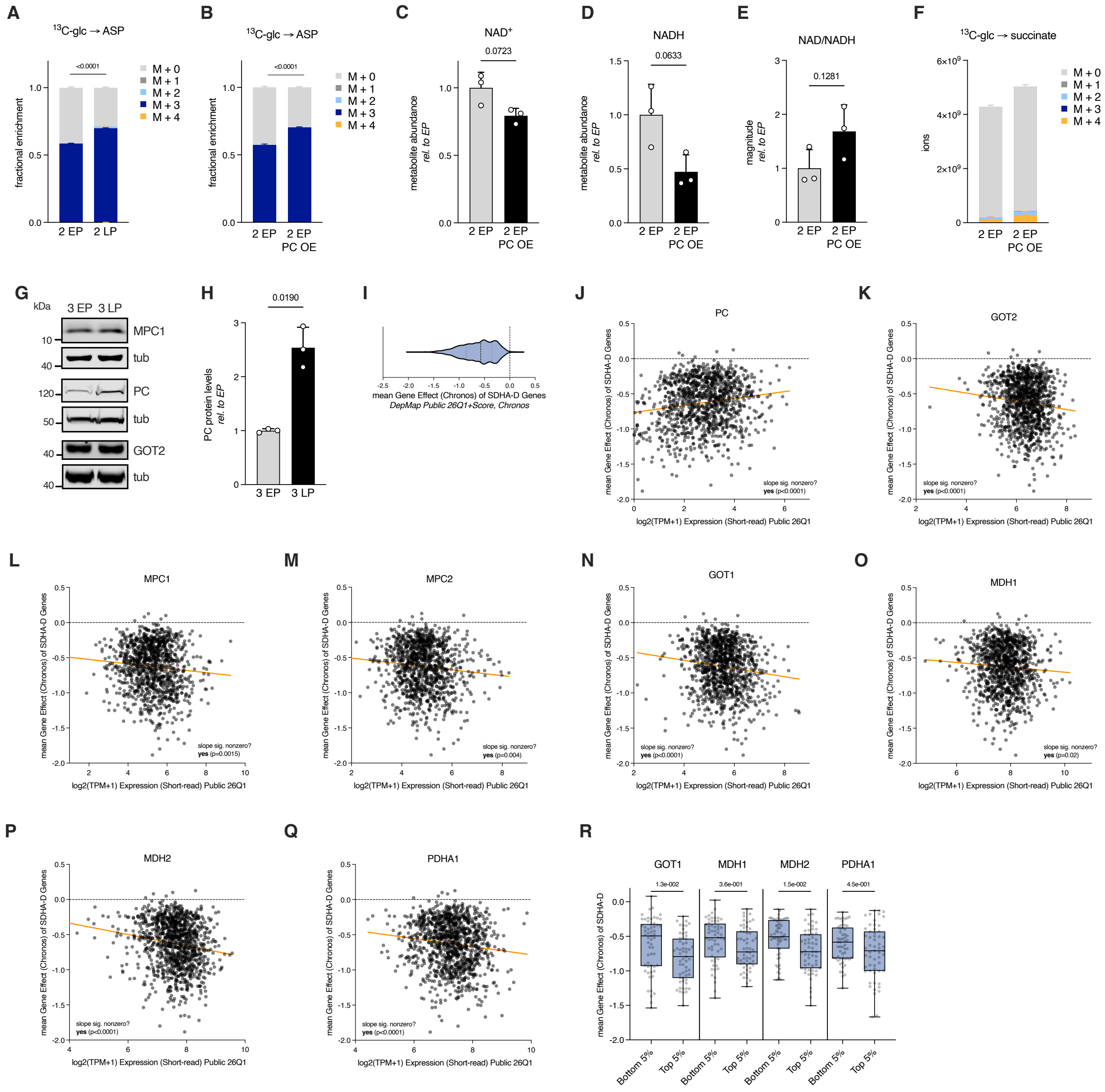

Figure S4. related to Figure 4.

(A) Fractional isotopolog distributions (mean +/- S.D.) for aspartate in early passage (EP) or late passage (LP) SDHB-KO clone 2 following 24 hours of tracing in media containing U-^{13}C glucose. Statistical significance determined using an ordinary two-way ANOVA and Sidak's multiple comparisons test with a single pooled variance. (n=3)

(B) Identical setup as in (A), comparing 2 EP and PC-overexpressing 2 EP (2 EP PC OE) (n=3)

(C) Relative NAD^+ levels (mean +/- S.D.) of 2 EP and 2 EP PC OE 24 hours after media change. Levels are normalized to 2 EP. Statistical significance determined using Welch's t-test. (n=3)

(D) Identical setup as in (C), measuring NADH levels. (n=3)

(E) Identical setup as in (C), measuring relative whole-cell NAD^+/NADH. (n=3)
(F) Absolute isotopolog distributions (mean +/- S.D.) for succinate in 2 EP and 2 EP PC OE following 24 hours of tracing in media containing U-^{13}C glucose. (n=3)
(G) Representative western blots showing levels of MPC1, PC, GOT2, and tubulin loading controls in early passage (EP) and late passage (LP) SDHB KO clone 3.
(H) Quantification of PC protein levels from 3 EP/LP using western blotting. Statistical significance determined using Welsh's t-test. (n=3) Quantification blot available in Source Data.
(I) mean SDHA-D gene effect scores for all cell lines in DepMap 26Q1
(J) mean SDHA-D gene effect scores for cell lines in DepMap 26Q1 plotted against relative expression of PC, fitted with linear regression (n=1140 cell lines)
(K) Identical setup as in (J), plotting against GOT2 expression (n=1140 cell lines)
(L) Identical setup as in (J), plotting against MPC1 expression (n=1140 cell lines)
(M) Identical setup as in (J), plotting against MPC2 expression (n=1140 cell lines)
(N) Identical setup as in (J), plotting against GOT1 expression (n=1140 cell lines)
(O) Identical setup as in (J), plotting against MDH1 expression (n=1140 cell lines)
(P) Identical setup as in (J), plotting against MDH2 expression (n=1140 cell lines)
(Q) Identical setup as in (J), plotting against PDHA1 expression (n=1140 cell lines)
(R) Mean SDHA-D DepMap gene effect scores for the top and bottom 5% of expressors of the indicated metabolic genes. Statistical significance determined using an ordinary one-way ANOVA and Sidak's multiple comparisons test with a single pooled variance. (n=57)

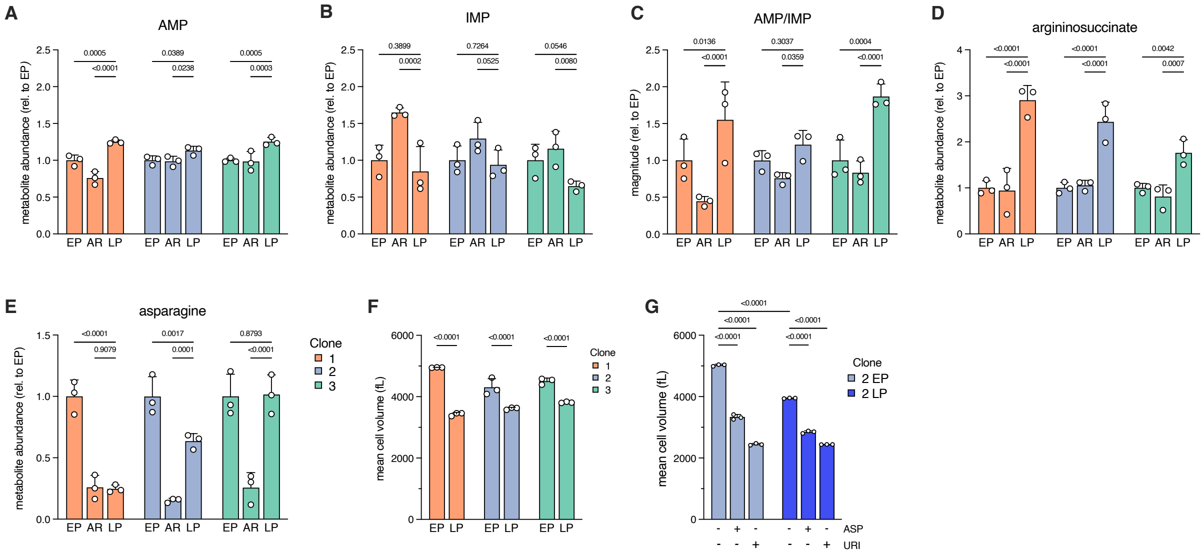

Figure S5. related to Figure 5.

(A) Relative adenosine monophosphate (AMP) levels (mean +/- S.D.) of early passage (EP), aspartate reared (AR), and late passage (LP) SDHB knockout clones 1-3. Levels are normalized to each corresponding EP clone. Statistical significance determined using an ordinary two-way ANOVA and an uncorrected Fisher's LSD with a single pooled variance (n=3)

(B) Identical setup as in (A), quantifying inosine monophosphate (IMP) levels (n=3)

(C) Identical setup as in (A), quantifying the AMP/IMP ratio (n=3)

(D) Identical setup as in (A), quantifying argininosuccinate levels (n=3)

(E) Identical setup as in (A), quantifying asparagine levels (n=3)

(F) Mean per-cell volumes of EP/LP SDHB KO clones 1-3. Statistical significance determined using an ordinary two-way ANOVA and an uncorrected Fisher's LSD with a single pooled variance (n=3)

(G) Mean per-cell volumes of EP/LP SDHB KO clone 2 supplemented with 20 mM aspartate (ASP) or 500 μM uridine (URI). Statistical significance determined using an ordinary two-way ANOVA and Tukey's multiple comparisons test with a single pooled variance (n=3)
